# Gout and Patellar Tendon Tears: An Uncommon Intersection

**DOI:** 10.7759/cureus.82930

**Published:** 2025-04-24

**Authors:** Amirzeb Aurangzeb, Vanessa Tan Wei Shen, Dinesh Sirisena

**Affiliations:** 1 Orthopaedic Surgery, Changi General Hospital, Singapore, SGP; 2 Sports Medicine, Ministry of Health Holdings, Singapore, SGP; 3 Sports Medicine, National University Health System, Singapore, SGP

**Keywords:** gout, knee, ligament, patella, tendon tear

## Abstract

Patellar tendon (PT) tears are a rare cause of anterior knee pain and are usually classified as either partial or complete, with various potential etiologies. We present a unique case of a PT tear in a young male patient, precipitated by a history of gout. This case highlights the uncommon intersection of PT tears and gouty arthritis involving the entheses.

The patient was a 36-year-old man of Indian origin who presented with a two-week history of atraumatic right knee swelling, with no identifiable precipitating factors aside from a known history of gout flares. Clinical examination revealed swelling and joint line tenderness in the right knee, though the overall assessment was otherwise unremarkable. In this paper, we explore management options for patients presenting with this rare manifestation of gout leading to a PT tear.

This case highlights the importance of early assessment through thorough history-taking, physical examination, utilization of appropriate investigation modalities, and expedited treatment to facilitate a favorable outcome. Early intervention enabled a swift recovery and return to sports for the patient. It also serves as a valuable reminder for clinicians to consider PT tears in the differential diagnosis of anterior knee pain and to investigate potential causes of such tears, which may influence subsequent management.

## Introduction

Patellar tendon (PT) tears are an uncommon cause of anterior knee pain, typically occurring in adults under the age of 40 [1,2]. These injuries may be partial or complete, with complete ruptures classically presenting as patella alta and an inability to actively extend the knee due to disruption of the extensor mechanism [2]. Although trauma is the most common cause, underlying tendon degeneration, whether from overuse, systemic disease, or metabolic conditions, can predispose individuals to rupture. Gout is the most prevalent form of inflammatory arthritis in males [3]. In the knee joint, it typically results in intra-articular crystal deposition affecting structures such as the menisci, popliteus tendon, cruciate ligaments, and quadricep tendons [3,4]. However, involvement of the entheses (tendon or ligament insertion sites) is exceedingly rare, with only a few cases documented in the literature [3,5,6].

This case describes a 36-year-old Indian male patient with gout who sustained a PT tear, raising the possibility that chronic gouty inflammation or crystal deposition may have contributed to tendon weakening and subsequent rupture. Given the rarity of both conditions, this case highlights an unusual but clinically important association.

## Case presentation

Our patient presented with a two-week history of atraumatic right knee pain. He was involved in a football game before the knee pain started, but there was no particular trigger during the football game itself, and he was able to jump and land on his feet after the match. However, suprapatellar and anterior knee pain started two days after the game. This resulted in knee instability, which worsened with walking and stair-climbing. He had a history of gout and is currently on diet control. He did not have any other medical problems and was not on any long-term medications. Significantly, he was a previous kidney donor. There was no known family history of inflammatory arthritis. The knee symptoms were affecting his work in civil service and his active lifestyle of playing football, running, and cycling.

**Figure 1:**
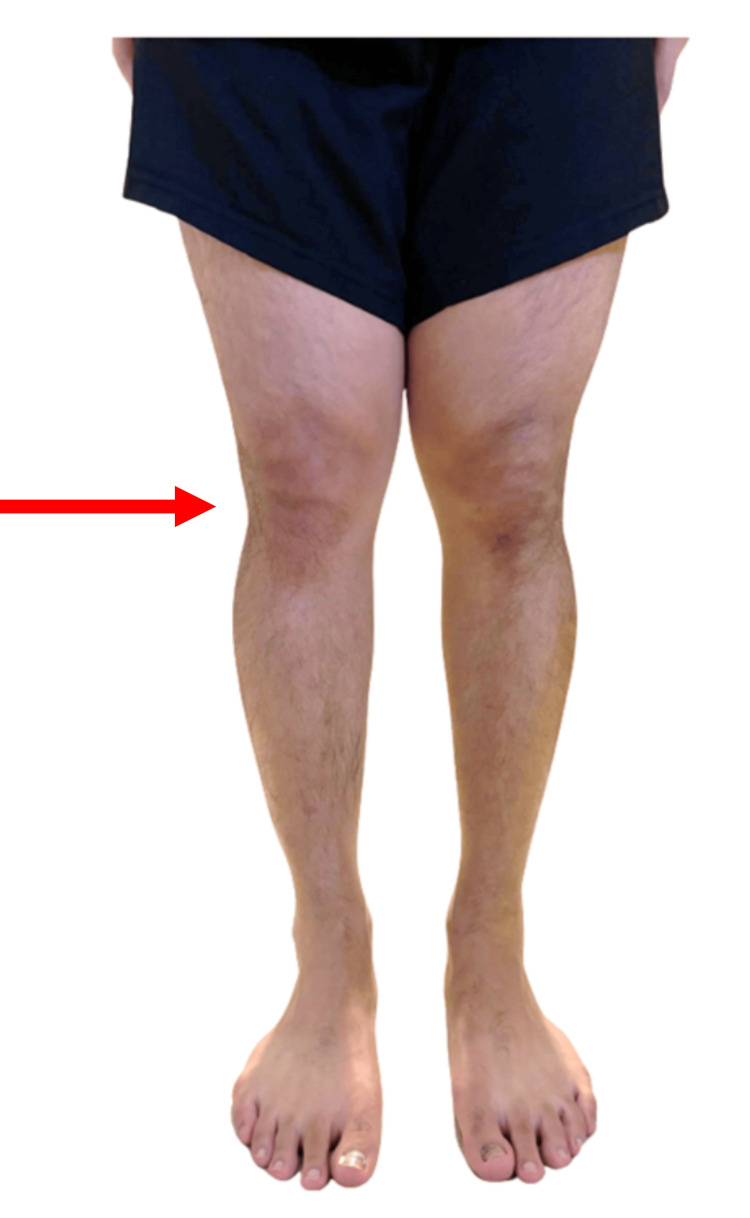
Anterior view of the bilateral knees of the patient during the initial presentation

On examination, he had no appreciable swelling in the right knee as seen in Figure 1. There was pain and tightness on squatting exercises, and the range of movement was reduced to 0-120 degrees. There was tenderness on palpation over the joint lines and PT, but assessments of the medial collateral, lateral collateral, anterior cruciate, and posterior cruciate ligaments were all negative. McMurray’s test was positive medially. Clinical examination of his left knee was unremarkable.

**Figure 2:**
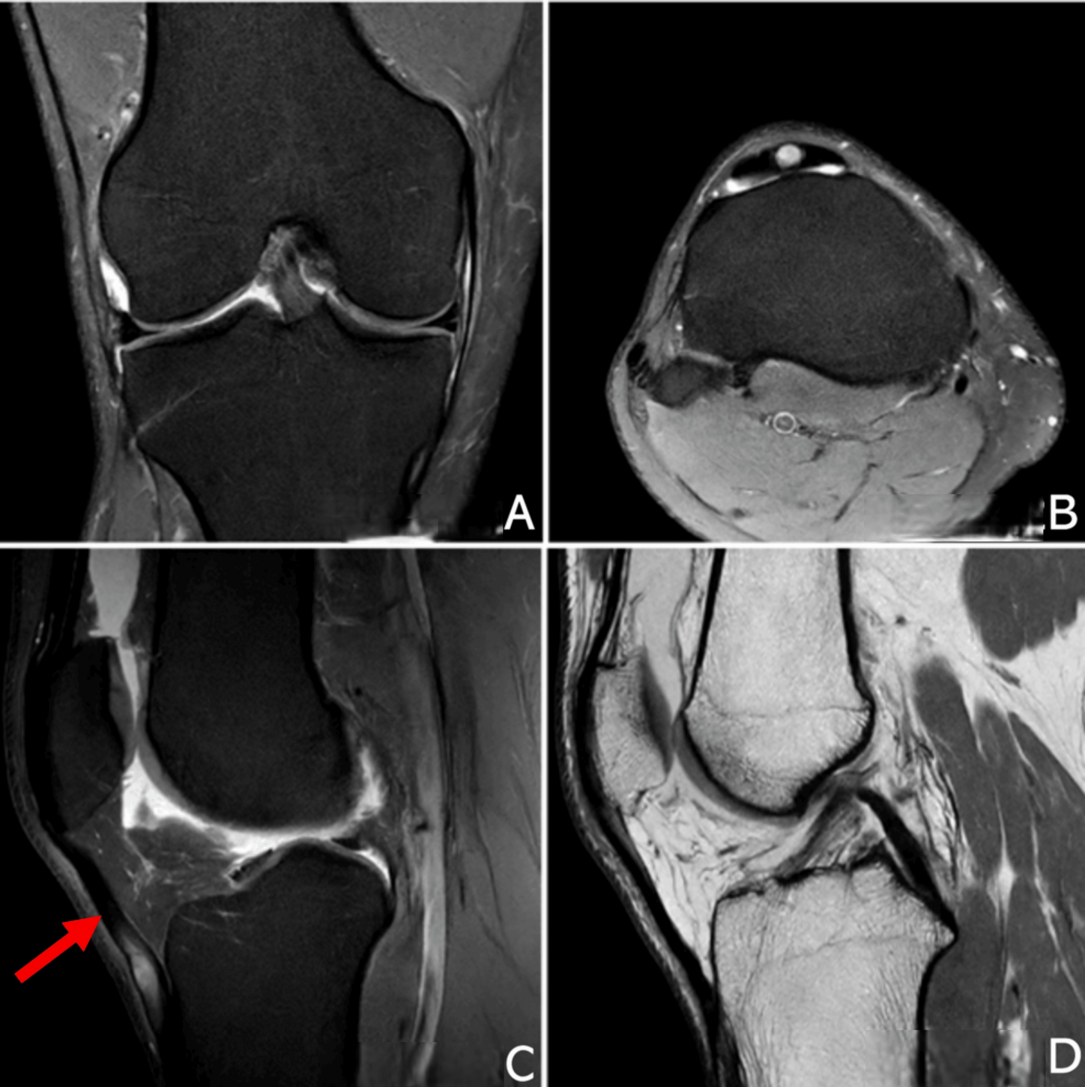
MRI T2-weighted images in the coronal (A), axial (B), and sagittal (C) views and T1 sagittal (D) view The image shows a patellar tendon intrasubstance tear with posteromedial meniscocapsular junction sprain without separation; intact anterior and posterior horns, body, and root attachments of the medial meniscus; and diffuse cartilage thinning on the weightbearing surfaces of the femoral and tibial condyles, without focal chondral ulcers, subchondral bony edema, osteochondral loose bodies, subchondral fracture line, and focal patellofemoral chondral ulceration/delamination. There was no evidence of lateral meniscal tear, and both cruciate ligaments are intact.

**Figure 3:**
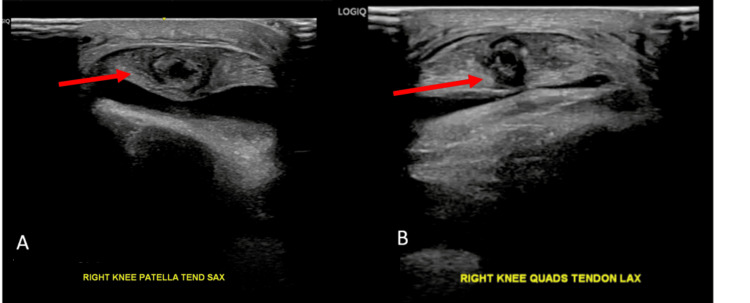
Ultrasound images of the right knee showing (A) hypoechogenic changes within the substance of the PT in keeping with an intrasubstance tear and (B) a hypoechogenic compressible substance seen on the ultrasound, which was eventually aspirated and had a chalky appearance

**Figure 4:**
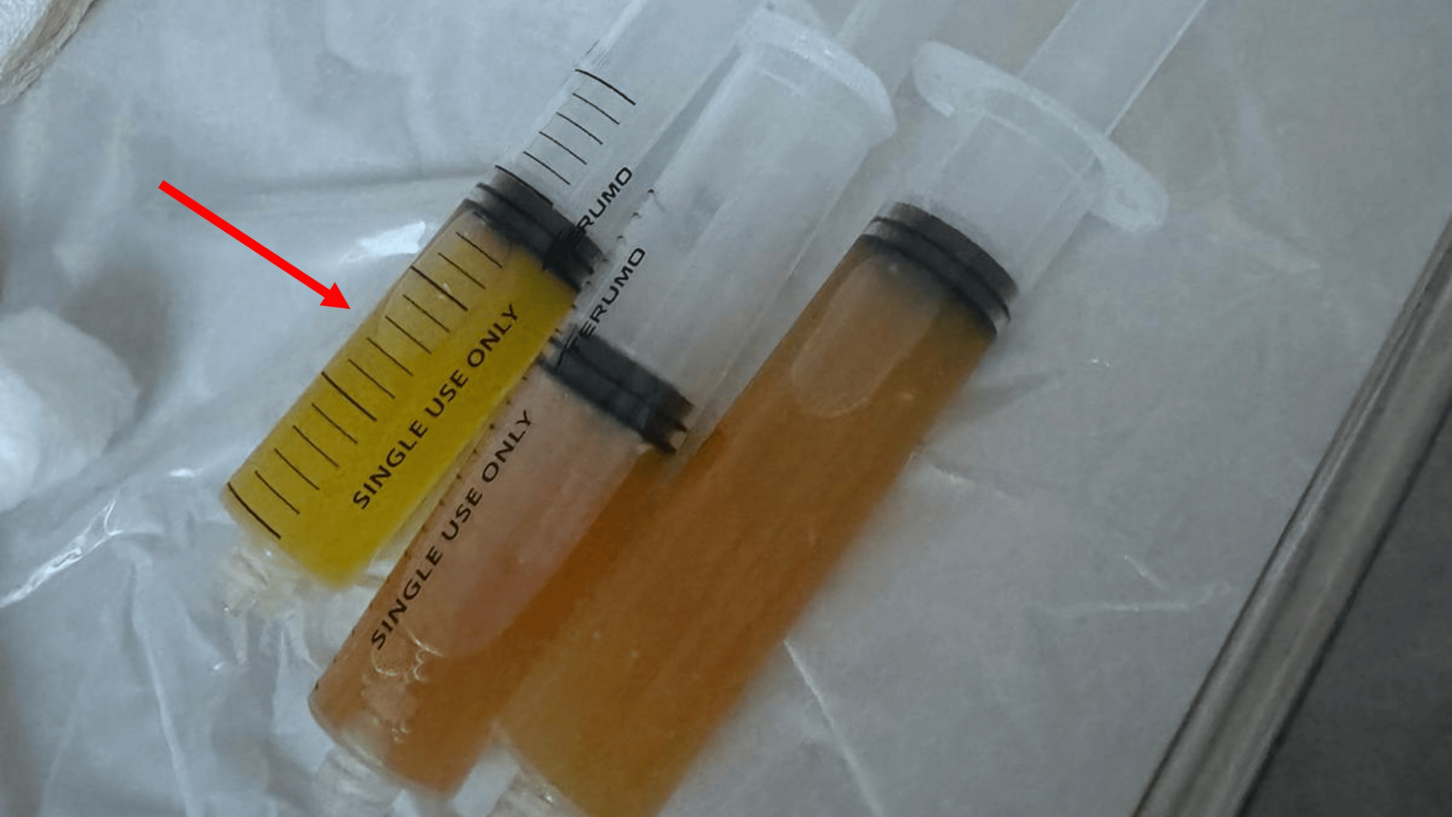
Clinical photograph of the cloudy and chalky aspirate from the right knee

Magnetic resonance imaging (MRI) scan of his right knee (Figures 2A-2D) revealed a PT intrasubstance tear, a posteromedial meniscocapsular junction sprain (without separation), and diffuse cartilage thinning. Due to the ongoing pain over the PT, the knee was offloaded with crutches, and an external knee brace was applied, limiting the range from 0 to 30 degrees. After 72 hours, there was minimal improvement in the pain over the PT. Ultrasound examination of the knee (Figures 3A, 3B) confirmed a compressible substance within the intrasubstance tear. On aspiration, a chalky gouty aspirate was extracted (Figure 4). A single platelet-rich plasma (PRP) injection was performed concomitantly for symptom control.

**Figure 5:**
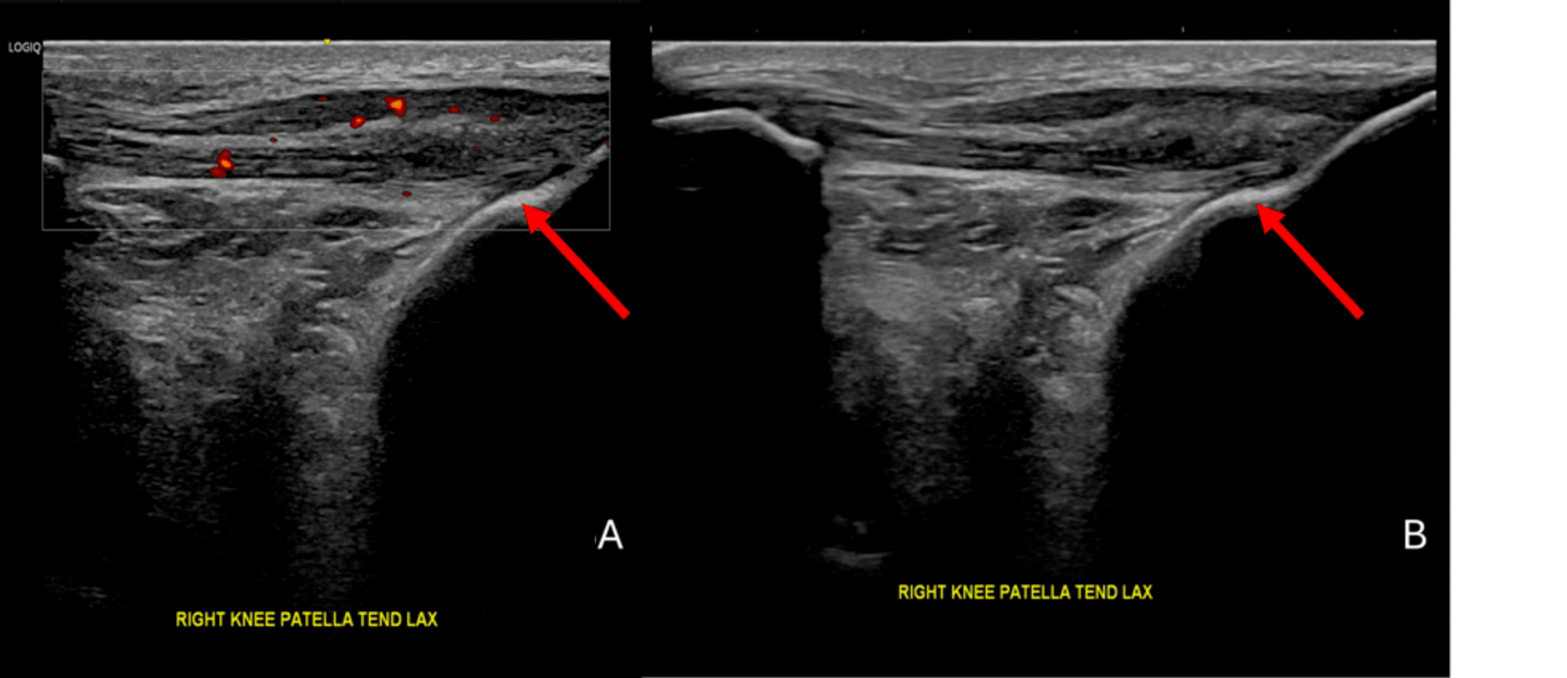
(A, B) Ultrasound images of the right knee showing healing of the patellar tendon with a hyperechogenic change in the tendon that was not compressible

Monosodium urate monohydrate crystals were found in the joint aspirate. Due to concurrent pain in the right foot's fifth toe, a one-week course of oral colchicine was commenced with oral analgesia (non-steroidal anti-inflammatory drugs (NSAIDs)) with advice on icing and compression of the knee. With this mode of treatment, his symptoms improved within a week. A repeat ultrasound scan of the knee (Figure 5) demonstrated tendon healing, and his knee range of movement was increased to 60 degrees using the knee brace. Serum uric acid was done at the point of review, and the three-month mark was noted to be in the range of 8.5-10 mg/dL (normal range: 4.00-8.20). Regular oral allopurinol was then commenced for the patient. A repeat serum uric acid after two months found a decrease to 7.23 mg/dL. The patient had also transitioned to a low-purine diet to aid in the general control of his gout symptoms. Overall knee symptoms had also resolved at this point. He did not have any knee pain or instability, which allowed him to successfully return to cycling without discomfort.

## Discussion

This case is particularly notable for two reasons. First, PT tears are relatively rare in general [1,4,5]. Second, gout typically affects the intra-articular aspects of the joint rather than the enthesis [3]. Thus, this represents a rare combination of gouty crystal deposition in the PT leading to tendon rupture. 

Gout results from hyperuricemia and is characterized by recurrent acute attacks of severe pain or chronic inflammation with tophaceous deposits, most commonly involving the Achilles tendon and flexor tendons of the hand [6]. In the knee, gout rarely affects the PT; instead, it more frequently involves the menisci, cruciate ligaments, and quadriceps tendon [6,7]. Current literature contains only a limited number of case reports describing gouty involvement of the PT [3,4,5].

PT injuries are usually associated with knee trauma; however, additional risk factors that predispose to tendon weakening include systemic disorders (autoimmune illnesses or chronic renal insufficiency), chronic local stress, and steroid administration [2].In this case, the patient had no significant trauma or conventional risk factors, suggesting that gout was the primary contributor to the tendon rupture. Although MRI identified fluid within the tendon, the echogenic characteristics of this fluid were more clearly appreciated on ultrasound. The hypoechoic, compressible substance was subsequently confirmed to be monosodium urate crystals upon aspiration. Indeed, aspiration and synovial fluid analysis remain essential diagnostic tools in such cases, particularly for confirming gout [8,9].

Ultrasound imaging in the outpatient setting proved useful in this case; not only did it support the diagnosis, but it also ensured accurate delivery of treatment and provided a cost-effective means of monitoring tendon healing over time. Management focused on addressing both the underlying gout and the PT injury, which were responsible for the patient’s acute knee pain and instability. PT was managed via immobilization and the use of a single ultrasound-guided PRP injection, which accelerated recovery [2]. The immobilization and progressive increase in movement helped alleviate pain symptoms while protecting the PRP-treated tendon. This combined approach ensured that there was a continuous progression and eventually a positive outcome with an eventual return to sports.

In the acute phase, gout is treated with oral medications such as NSAIDs and colchicine. Once the acute symptoms have settled, treatments such as allopurinol and probenecid can be commenced with preventative goals in mind [5]. However, the presence of gouty tophi and persistent pain or loss of function after failed conservative therapies may prompt the need for surgical intervention [5]. In this case, the gout responded well to medical therapy, with an improvement of symptoms and a decrease in the serum uric acid level. In a different scenario where the PT has ruptured, an early surgical repair would be necessary to preserve extension function [2,10]. 

We performed a detailed literature search on PubMed database to review other articles on similar topics to demonstrate the rarity of the case presentation. The search interval was set up to October 1, 2024. Keywords of “patellar tendon” and “gout” were used with English language as a search filter.

A total of 31 articles were identified for initial screening. Three articles were not available in English and thus excluded. Twenty-eight articles underwent a title screening process. Eighteen records were excluded based on the title screening process, as they were not relevant to the topic discussed. The remaining 10 articles underwent an abstract screening process, and four articles were further removed due to irrelevance to the topic. The remaining six articles are summarized in Table 1, with a relevant summary of each study as well as relevant learning outcomes from the study which may be useful along the topics of PT and gout.

**Table 1:** Summary of relevant literature

S/N	Title	Main author	Country	Year	Study design	Number of cases	Summary	Learning points
1	Tendon involvement in patients with gout: an ultrasound study of prevalence	Ventura-Ríos et al. [7]	Multi-national	2016	Cross-sectional study	80	Gout commonly affects tendons in the lower limbs, including the patellar tendons. Ultrasound is useful in detecting the presence of intra-tendinous tophi.	Gouts can affect the entheses such as the patella. However, there is no mention of gout leading to patellar tendon tears in such patients, only the presence of tophi itself.
2	Diagnosis and treatment of patellar tendon gouty tophus: a case report	Bouras et al. [6]	United Kingdom	2019	Case report	1	A 53-year-old manual worker who presented with right knee pain and stiffness with a history of gout. Eventually found to have a gouty tophus on MRI and underwent surgical excision of the tophus.	The presence of gouty tophus in the patellar tendon can lead to knee pain that is difficult to diagnose and manage. Surgical intervention may be necessary for patients with symptomatic gouty tophi in the knee if medical management has proved unsuccessful. No mention about the presence of patellar tendon tears in this paper.
3	Gouty destruction of a patellar tendon reconstruction and novel revision reconstruction technique: a case report	Edge et al. [3]	United States	2024	Case Report	1	A 56-year-old female patient with a known history of gout who presented with difficulty in weightbearing in the left leg and extending her left knee. She had a known history of tophaceous gout and left patellar tendon rupture with multiple previous revision surgeries done. The patient was subsequently treated with a novel allograft reconstruction technique.	This case report shows how gouty arthritis can lead to the rupture of patellar tendons due to severe gouty infiltration of the tendons, which needs multiple surgeries with increasing complexity.
4	Intratendinous tophaceous gout imitating patellar tendonitis in an athletic man	Gilliland et al. [5]	United States	2011	Case Report	1	A 45-year-old man with left anterior knee pain and enlarging mass of the patellar tendon. Surgical excision of the mass showed results consistent with that of a gouty mass.	Gouts were not considered a differential in the initial management. Gouty tophi in the patella is known, but minimal literature exists on athletes presenting with gouty patellar tendinopathy that mimics that of a jumper’s knee. No mention of the presence of patellar tendon tears in this paper.
5	Unusual presentation of gout: intratendinous tophus in the patellar tendon	Cimşit et al. [9]	Turkey	2015	Case Report	1	A 34-year-old male patient with a known history of gout who presented with pain and acute swelling in his extremities. Thickening of the patellar tendon seen on the X-ray.	The patella tendon is a possible site of involvement for gout, but there is minimal literature describing the presence of this in patients with patellar tendonitis. No mention of the presence of patellar tendon tears in this paper.
6	Enthesopathy and tendinopathy in gout: computed tomographic assessment	Gerster et al. [4]	Switzerland	1996	Case Report	3	A 69-year-old male patient with a known history of gout had inflammation over the left prepatellar bursa and patella tendon. Left knee swelling was noted with tenderness over the tibial insertion of the patella. CT scan of the knee showed round and oval opaque masses within the patellar tendon. The two other cases did not have patellar tendon involvement.	Computed tomography (CT) scans are useful in the imaging workup for patients with suspected gouty involvement in the entheses. No mention of the presence of patellar tendon tears in this paper.

Notably, only one case report specifically addressed the presence of PT tears in a patient with a known history of gout, while the majority of the other studies demonstrated a specific focus on patellar tendinopathy instead [3]. Thus, this demonstrates the rarity of the injury seen in this patient.

## Conclusions

This case illustrates two rare clinical entities, PT tear and gouty entheseal involvement, in a patient who achieved excellent recovery with a combination of medical management, immobilization, and a single PRP injection. It underscores the importance of early assessment, targeted investigation, and prompt intervention while also emphasizing the need to explore underlying systemic pathologies in cases of tendon rupture. Despite the unusual presentation, this multimodal approach facilitated a rapid and successful return to sports and physical activity. Furthermore, it serves as a critical reminder for clinicians to include PT tears in the differential diagnosis of anterior knee pain, particularly in active adults, and to investigate potential predisposing factors, including inflammatory conditions like gout, even in atypical presentations. By maintaining a high index of suspicion for both mechanical and systemic contributors, clinicians can optimize outcomes in similar cases.
